# Feedborne Mycotoxins Beauvericin and Enniatins and Livestock Animals

**DOI:** 10.3390/toxins13010032

**Published:** 2021-01-05

**Authors:** Ludmila Křížová, Kateřina Dadáková, Michaela Dvořáčková, Tomáš Kašparovský

**Affiliations:** 1 Department of Animal Breeding, Animal Nutrition and Biochemistry, Faculty of Veterinary Hygiene and Ecology, University of Veterinary and Pharmaceutical Sciences, 61242 Brno, Czech Republic; krizoval@vfu.cz; 2 Department of Biochemistry, Faculty of Science, Masaryk University, 61137 Brno, Czech Republic; k.dadakova@mail.muni.cz (K.D.); 451832@mail.muni.cz (M.D.)

**Keywords:** beauvericin, enniatins, minor mycotoxins, feed, metabolism, carry-over

## Abstract

Mycotoxins are secondary metabolites produced by several species of fungi, including the *Fusarium*, *Aspergillus*, and *Penicillium* species. Currently, more than 300 structurally diverse mycotoxins are known, including a group called minor mycotoxins, namely enniatins, beauvericin, and fusaproliferin. Beauvericin and enniatins possess a variety of biological activities. Their antimicrobial, antibiotic, or ionoforic activities have been proven and according to various bioassays, they are believed to be toxic. They are mainly found in cereal grains and their products, but they have also been detected in forage feedstuff. Mycotoxins in feedstuffs of livestock animals are of dual concern. First one relates to the safety of animal-derived food. Based on the available data, the carry-over of minor mycotoxins from feed to edible animal tissues is possible. The second concern relates to detrimental effects of mycotoxins on animal health and performance. This review aims to summarize current knowledge on the relation of minor mycotoxins to livestock animals.

## 1. Introduction

Mycotoxins are a structurally diverse group of mostly low-molecular-weight compounds. Their structures range from single heterocyclic rings to irregularly arranged rings of six to eight members and their molecular weights are usually less than 1000 Da. Therefore, they do not induce any response in the human immune system [1]. Mycotoxins are produced mainly by the secondary metabolism of certain filamentous fungi, which grow under specific temperature and humidity and cause serious risks for human and animal health. As secondary metabolites, instead of playing a role in growth and normal metabolism of the fungus, many mycotoxins are involved in pathogenesis or in competing with other organisms [1,2].

Many of the toxigenic fungi are ubiquitous and, in some cases, have a conjunction with food and feed production. From these, the most common toxigenic species belong to four genera: *Fusarium*, *Aspergillus*, *Penicillium*, and *Alternaria* [3]. *Fusarium* and *Alternaria* usually produce mycotoxins before harvest or in freshly harvested products, whereas *Aspergillus* and *Penicillium* species represent a higher risk during drying and storage of food and feed products [1,3]. *Fusarium* genus includes over 90 described species and is responsible for the production of some of the most important classes of mycotoxins: trichothecenes, fumonisins, and zearalenones. Moreover, this genus produces less studied mycotoxins called minor or emerging mycotoxins: fusaproliferin, beauvericin (BEA), enniatins (ENs), and moniliformin. The toxicity of the toxins produced by *Fusarium* varies greatly depending on the toxin and the target organism [3,4]. The most important species that produce these toxic metabolites are *Fusarium proliferatum*, *Fusarium subglutinans*, *Fusarium moniliforme*, and *Fusarium avenacum*, involved in crop diseases, such as stalk and maize ear rot disease [5].

To date, more than 300 mycotoxins have been identified, and research is focused mainly on those that have been proven to have diverse health effects on humans and animals, like teratogenicity, carcinogenicity, and mutagenicity [6,7]. The exposure of humans to mycotoxins occurs either directly through the consumption of contaminated plant foods (e.g., cereals) or indirectly through the intake of animal-derived products (e.g., milk and eggs) that origin from animals fed with contaminated diets [6]. From the perspective of livestock breeding and nutrition, mycotoxins in feedstuffs are of dual concern. First one is connected with the safety of animal-derived food and is related to occurrence of mycotoxins in feed and their (partial) carry-over from feed to edible animal tissues such as milk, eggs, or meat. The occurrence of mycotoxins in food can be legislatively monitored (presence of aflatoxin M1 in milk [8]). The second one is connected with detrimental effects of mycotoxins on animal health and performance. Such detrimental mycotoxins include deoxynivalenol (DON), fumonisins, ochratoxin A, and zearalenone (ZEA). Most, but not all, of these mycotoxins are produced by the *Fusarium* species. Although these mycotoxins are significant contaminants when entering the food chain directly via food of plant origin, they are not considered relevant in food of animal origin because their carry-over from feed to animal-derived food products is negligible [9,10,11,12]. However, *Fusarium* species are also responsible for the production of minor mycotoxins, namely, enniatins and beauvericin, which are currently in the center of interest because of the wide range of their biological activities, as described in details bellow.

## 2. Beauvericin

**Figure 1:**
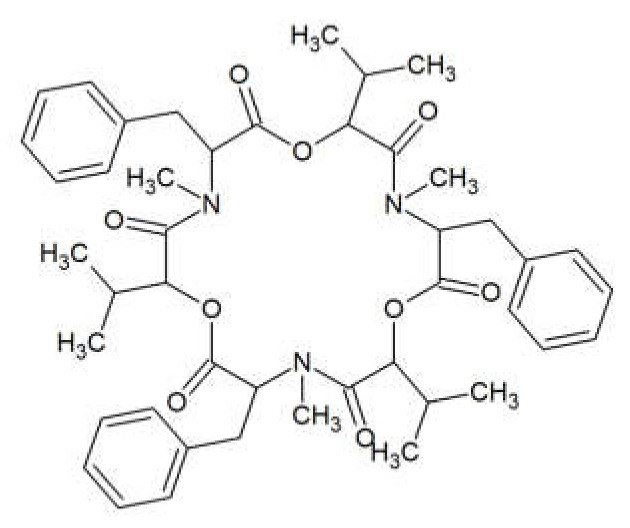
Beauvericin structure.

Beauvericin (BEA) is a cyclic lactone trimer, which contains an alternate sequence of three N-methylphenylalanyl and three D-α-hydroxyisovaleryl residues (Figure 1). It was first isolated from the fungus *Beauverina bassiana*, an insect pathogen [13]. The first *Fusarium* species identified to produce BEA was *Fusarium subglutinans* [14]. Subsequently, other *Fusarium* species such as *Fusarium bulbicola*, *Fusarium denticulatum*, *Fusarium lactis*, *Fusarium phyllophillum*, *Fusarium pseudocircinatum*, and *Fusarium succisae* have been proven to produce BEA [15].

BEA possesses insecticidal and phytotoxic properties and is involved in the etiology of insect and plant diseases caused by the producer fungal strains [16]. The antimicrobial and antibiotic activities of BEA have been tested on human or mammalian intestinal bacteria (see Table 1). BEA also showed endocrine disrupting antagonistic effects at the androgen receptor [17]. It also acts on cellular level as an enzyme inhibitor [18], and as a compound inducing oxidative stress. BEA eases apoptosis, interferes with smooth muscle contraction, impedes with steatosis caused by the storage of cholesterol in liver cells, and according to various bioassays, it is believed to be toxic. Nevertheless, it was discovered that cytotoxicity of BEA depends on the dose, length, and also way of exposure [19,20], because it is able to penetrate to the body through the skin, although its permeation is relatively low [21].

Furthermore, the effect of BEA on human and animal health might not be just negative, BEA was also proven to have several positive qualities such as antifungal [22], antiviral [23] or antibiotic effect. The antibiotic effects of BEA were tested on the following bacterial species including those from GI tract: *Bacillus cereus*, *Bacillus mycoides*, *Bacillus pumilis*, *Bacillus sphaericus*, *Bifidobacterium adolescentis Clostridium perfringens*, *Escherichia coli*, *Enterococcus faecium*, *Eubacterium biforme, Listeria monocytogenes*, *Paenibacillus alvei*, *Paenibacillus azotofixans*, *Paenibacillus macerans*, *Paenibacillus macquariensis*, *Paenibacillus pabuli*, *Paenibacillus productus, Paenibacillus pulvifaciens*, *Paenibacillus Validus, Peptostreptococcus anaerobius, Pseudomonas aeruginosa*, *Salmonella enterica*, *Shigella dysenteriae*, *Yersinia enterocolitica*, and two strains of *Staphylococcus aureus*, using microbial bioassay techniques [24,25,26]. The highest activity was observed for *C. perfringens* with a minimum inhibitory concentration (MIC) of 1 ng per disc, followed by *S. enterica* (MIC = 10 ng per disc) and *B. pumilus* together with *L. monocytogenes* (MIC = 100 ng per disc). Generally, Gram-positive bacteria were more inhibited than Gram-negative ones. Furthermore, BEA, which acts as an inhibitor of activated T cells, is a possible drug candidate for the colon inflammation treatment [27].

## 3. Enniatins

**Figure 2:**
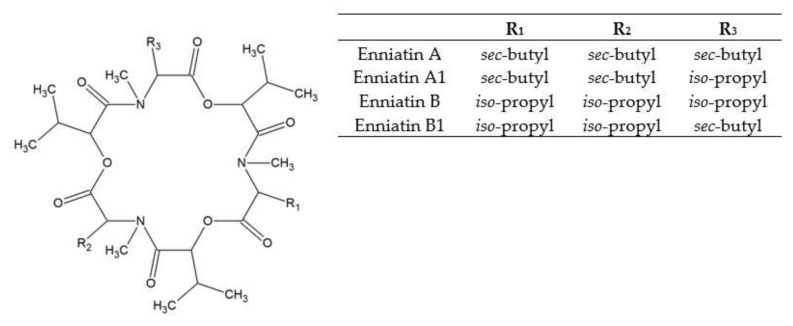
Enniatin structure.

Enniatins (ENs) were discovered in the cultures of *Fusarium orthoceras*, later renamed *Fusarium oxysporum* [28]. ENs represent a large group of related mycotoxins with the structure of cyclic hexadepsipeptides, comprised of D-α-hydroxy-isovaleryl-(2-hydroxy-3-methylbutanoic acid) and N-methylamino acid residues linked with peptide bonds and intra-molecular ester (lactone) bonds (see Figure 2). ENs of type A and B contain N-methyl-valine or N-methyl-isoleucine or the mixtures of these two amino acids [29]. Currently, 29 naturally occurring enniatin analogues are known [30] and seven of them (ENs A, A1, B, B1, B2, B3, and B4) have been found in cereals. ENs A, A1, B, and B1 are most frequently reported in foods and feeds [15]. ENs are produced by strains of some species of *Fusarium*, *Alternaria*, *Halosarpheia*, and *Verticillium* genera [31].

ENs are phytotoxic [32], antifungal (towards *Aspergillus flavus*, *A. parasiticus*, *A. fumigatus*, *A. ochraceus*, *Beauveria bassiana*, *Fusarium verticilloides*, *F. sporotrichioides*, *F. tricinctum*, *F. poae*, *F. oxysporum*, *F. proliferatum*, *Penicillium expansum*, and *Trichoderma harzianum*) [24], antiyeast (towards *Candida albicans*, *Trichosporum cutaneum*, and *Cryptococcus neoformans*) [33] and antibacterial (towards *Bacillus subtilis*, *Mycobacterium* spp., *Pseudomonas aeruginosa*, *Staphylococcus aureus*, *Escherichia coli*, and lactic acid bacteria) [34] and insecticidal agents [35]. The antimicrobial activities of ENs tested on human or mammalian intestinal bacteria are shown in Table 1.

ENs have cytotoxic activities that have been tested on several mammalian and cancer cell types such us Hep-G2 [36,38], Caco-2, HT-29 [36], MRC-5 [38] or CHO-K1 cells [39]. These studies gave proof of the potential cytotoxicity of ENs in mammal cell lines at quite low micromolar concentrations. Furthermore, synergistic effect of the combination of several individual ENs was observed [39]. It has been proved that ENs might even have an effect of genotoxicity. When eaten in larger doses, symptoms that are frequently occurring in transition cows include reduced rumen fermentation [40]. ENs are able to penetrate to the body through the skin and their permeation is higher than that of BEA with the highest permeation found in enniatin B (k (p, v) = 9.44 × 10–6 cm/h) [21].

ENs are also known as ionophores [41,42], antibiotics [24], and antimicrobial compounds [43,44] against human, animal, and plant pathogenic bacteria with no selectivity between Gram-positive and Gram-negative bacteria. Undeniable benefit of ENs is also an anti-helminthic effect [45]. Their biological activities may be explained by their ability to selectively increase the flux of alkali metal ions through biological membranes. Using the patch clamp technique in the inside-out mode, enniatin was shown to incorporate into the cell membrane, where it forms pores selective for cations [46]. Recent study suggested that EN B1 can destabilize the lysosome-associated membrane proteins 2 which results in the alkalinization of lysosomes and partial lysosomal membrane permeabilization [47]. In addition to their effect on cells, ENs exerts a hypolipidemic effect partly by inhibiting enzymes such as acyl-CoA: cholesterol acyl transferase (ACAT) and partly by reducing triglyceride synthesis and diminishing the free fatty acid pool in the cells. Furthermore, ENs inhibit 30,50-cyclo-nucleotide phosphodiesterase and can attach to calmodulin. Even though, ENs are currently used only to the local treatment of respiratory infections [46].

## 4. Presence of Beauvericin and Enniatins in Feedstuffs

In the EU, over 163 million tons of compound feeds are produced, accounting for approximately 50% of all feedstuffs [48]. In addition to the inclusion of compound feeds, cereal grains and by-products are consumed on farm as mixes or as a single ingredient, particularly to supplement forages for farm ruminants [49]. However, no details are available on the total amount of cereals (grains and by-products) used as feed, neither concerning the crops used (wheat, barley, oats, etc.), nor the farm animals (cattle, pigs, poultry, etc.). Oily products are also commonly used in animal feed. Contamination of *Fusarium poae* of beans has been proven [50], so there may be a possibility of BEA contamination, but there is little information available about contamination of this kind of feed. *Fusarium* species that produce mycotoxins can also infect other crops and their products, including grass, hay, and straw, which are animal food sources, and cause mycotoxicosis when fed directly to animals [51].

The growth of *Fusarium* spp. and the resulting mycotoxin content in feedstuffs can be affected by multiple factors such as environmental conditions (e.g., temperature and moisture conditions), geography and agricultural practices during various stages of production (e.g., tillage systems, pesticide treatment, and storage conditions) [3,52]. Because these conditions can vary between years, *Fusarium* toxins are expected to occur more frequently and at higher concentrations in years when weather conditions are favorable for fungal development [53]. Although the available data indicate a relationship between the local climate and mycotoxin concentrations, the interactions between the above mentioned factors are not yet thoroughly understood, and therefore toxin production cannot be predicted [3,52].

In last years, data on the occurrence of beauvericin (BEA) and enniatins (ENs) in livestock feedstuff have been reported. BEA and ENs are predominantly found in cereals (grains and products) [44,54,55,56,57,58,59]. However, they have also been detected in by-products from various types of industry [60,61] so their presence was logically noted also in compound feeds for livestock animals [62,63,64]. Further, they have been detected also in forage feedstuff [65,66,67,68,69] but the contamination differs between crops. In a total of 288 grain samples from Norway, the concentration of BEAs and ENs was significantly higher in wheat and barley than in oats [44,54]. Data from Sweden also confirm that wheat is more prevalent than oats, with the occurrence of BEA and ENs [70,71]. Other studies have shown that maize silage is more susceptible to contamination with various mycotoxins than grassland products [72]. Both maize and wheat silages frequently contain BEA and ENs [65,73,74]. In a study on European maize silages, BEA was found in 76% of the samples with median and maximal concentrations of 9 and 214 µg/kg, respectively [66]. ENs were found in more than 78% of the samples. The most abundant were enniatin B and B1 with median concentrations of 7 and 6 µg/kg, respectively, and maximal concentrations of 429 and 555 µg/kg, respectively [66].

Concentrations of BEA and ENs may further vary due to feed processing. Plants or grains can be either fed directly after harvest or further processed to be preserved. The most frequent strategies of conservation are drying of the grass and forages to produce hay, or utilizing spontaneous anaerobic lactate fermentation of maize, clover, grass, or forage to obtain silage and/or haylage. During ensiling, degradation or transformation of pre-harvest mycotoxins may also occur, as confirmed by several studies on enniatin B that found significantly lower concentrations in ensiled maize than in fresh maize [75,76]. This is supported by findings from food fermentation processes [77]. However, the stability of ENs and BEA during ensiling has not been extensively studied yet.

Furthermore, technological processing of grain, such as sorting, dehulling or peeling, can modify the content of ENs in feedstuffs because higher concentrations of EN B and B1 were found in small kernels compared to unsorted grain [78] and majority of EN B and B1 were associated with the bran or hulls with a much lower presence, or even absence in the groats or flour [77,79]. Partial degradation of BEA ranging from 43.0% to 87.6 % was also noted after a heat treatment in dependence on temperature (160–200 °C) and heating time (3 to 20 min) employed and on the composition of heated matrix, because some dietary components may have protective effects on BEA [80].

In addition, failure in feed processing methods (good sorting, proper storage conditions etc.) usually results in increased mycotoxin production [81,82,83]. In some cases, when feedstuffs or feedstuff supplements are produced as products of other technologies (e.g., dried distillers’ grains with solubles), the pollution levels of the mycotoxins contained in them depend both on the quality of the input material and on the technological process itself. For example, dilution or concentration of dry matter during the process was found to play a role [84,85].

Co-occurrence of ENs and BEA with other fusarium mycotoxins such as deoxynivalenol or nivalenol was often reported [55,86,87,88]; thus, not only the toxicity of individual mycotoxins, but also their possible synergistic, additive, or antagonistic effects on animals, should be taken into consideration. To our knowledge, these effects on various cells were described for combinations of different *Fusarium* mycotoxins, such as BEA, T-2 toxin (T-2) and DON; ENs A, A1, B, and B1; BEA, DON, enniatin B, FB1, T-2, and ZEA; T-2 toxin and enniatin B1 (recently reviewed by Smith et al. [89], Mallebrera et al. [90] and Prosperini et al. [91]), DON, enniatin B and alternariol [92]; DON, BEA, ZEA, enniatin A, A1, B, B1, alternariol, tentoxin, and mycophenolic acid [93] or BEA and ZEA derivates [94]. Studies on the acute toxicity of combinations of mycotoxins report ambiguous results, showing that the interaction depends on several factors like the cell models and doses tested. The co-occurrence of *Fusarium* mycotoxins probably increases their toxic effects compared to a single mycotoxin [93,95]. However, in another study, the co-occurrence of emerging mycotoxins did not change the toxicity of DON [96] or moniliformin [97].

EFSA calculated acute and chronic exposure of farm animals to BEA and ENs (the sum of ENs A, A1, B, and B1) using the mean and 95th percentile lower bound (LB) and upper bound (UB) occurrence data in cereals (grains and their products). Regarding the chronic exposure, ruminants and horses had the highest exposure to BEA (UB of 0.86 μg/kg b.w. per day) and poultry had the highest exposure to ENs (UB of 27.8 μg/kg b.w. per day). Regarding the acute exposure, poultry had the highest exposure to both BEA (1.89 μg/kg b.w. per day) and ENs (113 μg/kg b.w. per day). However, limits for BEA and ENs concentration have not been established, although their presence has been assessed by EFSA in feed at high levels (up to mg/kg or ppm) [98]. Furthermore, it should be pointed out that due to the lack of the data on BEA or ENs concentrations in forages, the exposures for ruminant livestock animals have been underestimated and real exposure to emerging mycotoxins is probably much higher.

## 5. Metabolism of Beauvericin and Enniatins

Knowledge on the metabolism of beauvericin (BEA) and enniatins (ENs) in animals are limited. Few data are available for ruminants, poultry, pigs, rabbits, guinea pigs, mice, and rats.

The oral absorption differs between the types of mycotoxins, including enniatin analogs, and animal species. For example, in pigs, the oral absorption of enniatin B was high [99], but that of enniatin A and enniatin A1 was low [100]. On the other hand, in broiler chicken, oral bioavailability of enniatin B and B1 was low [101], all resulting in large differences in plasma concentrations of studied mycotoxins. According to Devreese et al. [102], enniatin B might have the highest oral absorption, followed by enniatin B1, A1, A, and finally BEA.

In vitro and in vivo data indicate that after absorption, BEA and ENs are rapidly metabolized to a variety of uncharacterized metabolites. Due to rapid metabolism, only phase I metabolism is relevant and includes hydroxylation, carboxylation, and N-demethylation reactions [103]. These processes are best described for enniatin B. According to Ivanova et al. [104], the incubation of enniatin B with chicken liver microsomes resulted in the production of four hydroxylated metabolites, three carboxyl metabolites, and a novel metabolite that was not formed in corresponding human-derived incubations. On the other hand, the human liver microsomes produced other demethylated metabolites, showing differences in the metabolism of enniatin B between species. These results suggest that in chicken, oxidation is the principal biotransformation pathway. Furthermore, as phase I metabolites, deoxygenated enniatin B (the most prominent), mono-, and di-demethylated enniatin B were recently identified in liver and colon of mice [105] and mono- and dioxygenated enniatin B1 metabolites were found in chicken [101]. On the other hand, phase II metabolites, that is sulfated or glucuronidated forms of ENs were not detected in chicken [101] but they were found in rats [106]. To our knowledge, no information on the occurrence of ENs and/or BEA in biological samples of ruminants is available except of trace amounts of enniatin B (0.35 ng/mL) in the cow rumen fluid taken 3 h before morning feeding detected by Debevere et al. [72]. In their subsequent study [107] using a rumen simulation, over 70% of enniatin B was degraded during 48 h under physiological pH, whereas under the conditions of subacute rumen acidosis, the degradation was impeded. As a consequence, depending on the rumen conditions, part of enniatin B may pass to the intestine of the ruminants.

Furthermore, Manyes et al. [106] identified two enniatin A degradation products, probably produced by gut microflora, in duodenum, jejunum, and colon content of rats. These were a K ^+^ adduct of enniatin A with the loss of isoleucine (EN A + K-Ile) ^+^ and a hydroxyvaleric acid unit (EN A + K-HyLv) ^+^. In addition, the adducts formed between enniatin A and the diet macronutrients were detected in the intestinal digesta of rats. In the duodenum and jejunum compartments, an adduct was formed between enniatin A and two molecules of glucose (EN A + 2Glu-H_2_O) ^−^. This adduct was the only one that was detected also in the serum, suggesting its absorption. An adduct of enniatin A and two molecules of glucuronic acid (EN A + 2Gluc.Ac.) ^−^ was found in duodenum and an adduct with four glucose units (EN A + K + 4Glu) ^−^ was found in colon [106]. Role of gut microflora in metabolism of ENs and BEA is not clear. However, except of the above mentioned adducts several products of ENs and BEA bacterial degradation were recently identified, mostly sodium or potassium adducts of the mycotoxins with the loss of an amino acid, isovaleric acid or carboxylic group (see Table 2) suggesting that gut microflora may play an important role in metabolism of these compounds.

On the other hand, inhibitory effects of *Fusarium* mycotoxins on bacteria present and functionally important in digestive tract of livestock animals should be also taken into account. Such inhibition was reported for *Ruminococcus albus*, the methanogenic archaeon *Methanobrevibacter* sp., members of the genus *Lactobacillus*, *Bacillus,* or *Streptococcus* [112,113]. Studies on antibacterial and antibiotic activities of BEA showed the highest activity for *Clostridium perfringens* followed by *Salmonella enterica* and *Listeria monocytogenes* [24]. In the study of Castlebury et al. [26], some of the Gram-positive anaerobes (*Bifidobacterium adolescentis*, *Clostridium perfringens*, *Eubacterium biforme*, *Peptostreptococcus anaerobius*, and *Paenibacillus productus*) were inhibited by BEA.

An inhibitory effect of ENs on bacterial growth was reported for *Staphylococcus aureus*, *Clostridium perfringens*, and *Salmonella enterica* (enniatin B, [36]). Roig et al. [37] used the disc diffusion method to test ENs A, A1, A2, B, B1, and B4 against nine species of lactic acid bacteria, 22 *Saccharomyces cerevisiae* strains, and nine *Bacillus subtilis* strains. The most active was enniatin B1, followed by A1, contrary to ENs A and A2 (each active only against one of the *S. cerevisiae* strains). Inhibitory effects of ENs were proved in many other studies [114,115]. However, these mycotoxin activities against bacteria have been tested under laboratory conditions in pure cultures; therefore, it remains unclear how *Fusarium* emerging mycotoxins affect the actual microbial community in the rumen.

On the other hand, some bacteria that can be also classified as natural probiotics, such as *Lactobacillus* or *Bifidobacterium* sp., are being tested as detoxifying agents for their binding activities against mycotoxins to decrease their bioavailability after ingestion. Most of these bacteria were tested against major and more frequently occurring dietary mycotoxins such as aflatoxin [116], ZEA [117] or ochratoxin A [118]. However, some studies [111,119] found also significant reduction of ENs and BEA bioavailability when different strains of *Bifidobacterium*, *Lactobacillus,* or *Eubacterium* spp. were employed as probiotic strains.

Based on their rat study, Manyes et al. [106] suggested that the main site of ENs absorption is jejunum. After absorption, both ENs B and B1 were readily distributed to the tissues and found in serum and liver but it seems that the volume of the distribution differs between animal species [99,101]. Elimination of ENs B and B1 from the body seems to be rapid, as three days after withdrawal of mycotoxins, neither parent mycotoxins nor their metabolites were found in the liver [101]. On the other hand, eggs gathered three days after mycotoxin withdrawal were positive for both enniatin B and its hydroxylated metabolite [104]. The described routes of excretion are via urine and feces, but the rate of excretion is probably dose-dependent. After a single administration of ENs (mixture of ENs A, A1, B, B1 at the exposure level of 50 mg/kg) in rats, 5–10% of enniatin B was excreted to urine within 24 h post exposure with a major portion of enniatin B detected in the urine samples between 6 and 24 h samples [120]. On the other hand, after single administration of a mixture of ENs containing 1.19, 2.16, 1.03 and 1.41 mg/kg body weight of enniatin A, A1, B and B1, respectively, concentration of ENs in urine were below LOQ. However, all four EN analogs were detected in feces with maximum concentrations 6 h after administration [23]. Further studies are needed to specify the routes of ENs and BEA excretion in livestock animals and also check the presence of various phase I metabolites in excreta because demethylated, oxidated, hydroxylated and carbonylated metabolites were recently tentatively identified in human urine samples [121].

## 6. Occurrence of Beauvericin and Enniatins in Foods of Animal Origin

There is only limited information available on the carry-over of beauvericin (BEA) and enniatins (ENs) from feed to animal-derived food. However, their lipophilic properties may lead to their accumulation in some animal tissues. Indeed, BEA and ENs have been detected in the laying hens’ eggs, with the accumulation of these mycotoxins in the egg yolk, and in some tissues of turkeys and broilers [122] with the highest prevalence of EN B1 [123]. Several metabolites of EN B were detected in the serum and liver of broilers and in eggs of laying hens. The carry-over rate of BEA and ENs B and B1 from feed to the meat, liver, and skin of broilers and to laying hens’ eggs is low. The highest rates of BEA were 1.57% and 1.16% in the liver and skin of broilers, respectively, and 0.44% in laying hen eggs and those of ENs were 0.04% in broiler thigh muscle [124]. This finding suggests that residues of BEA and ENs from poultry contribute probably only marginally to the exposure of humans [98]. Several studies report that in farmed fish, the highest content of ENs was found in edible muscles and liver, but BEA was not detected in edible tissues [125,126]. However, no transfer of parent emerging mycotoxins from feed to fish was reported by Nácher-Mestre et al. [127].

The carry-over of these substances into milk may also be possible. Piatkowska et al. (2018) [128] detected low levels of EN B in 18 out of 20 samples of sheep milk with the average concentration of 7.8 ± 1.7 ng/kg. Carry-over of ENs and BEA from food to milk has been also documented in recent human studies [129,130,131], where low amounts of BEA (5.4.ng/L), EN A (20.1–51.1 ng/mL) and B (90.7–110.3 ng/mL) were detected. However, no data on occurrence of these emerging mycotoxins in bovine milk are available.

## 7. Conclusions

*Fusarium* fungi produce some of the most important classes of mycotoxins, but they are also responsible for the production of the so-called minor or emerging mycotoxins, enniatins (ENs), and beauvericin (BEA), which possess a wide range of biological activities. They are predominantly found in cereals and cereal-based products, but their occurrence in forages has also been reported. Their presence in feeds either alone or in combination with other mycotoxins represents a risk for animals and via entry to the food chain also a potential risk for humans because their carry-over to animal-derived products was proven.

Metabolism of ENs and BEA has been studied in monogastric animals, data on ruminants are limited. Based on the available data, it can be concluded that ENs and BEA are absorbed and rapidly metabolized to a variety of yet uncharacterized metabolites and that the course of metabolism differs between animal species. Further, gut microflora seems to play a significant role in the metabolism of these mycotoxins as well, but its role is not clear. Attention should also be focused on the possible synergistic, additive and/or antagonistic effects of emerging and other mycotoxins present in feeds that may result in unexpected health risks.

Based on the available data, the carry-over of ENs and BEA from feed to edible animal tissues is possible, but their concentration in animal-derived food is low. However, the ingestion of low doses of these toxic compounds in animal-derived food over long periods of time could increase overall long-time dietary exposure of humans to mycotoxins and could pose a health risk for consumers.

## Figures and Tables

**Table 1:** Antimicrobial effects of beauvericin and enniatins B and B1 on bacteria isolated from the human or mammalian intestinal tract.

**Beauvericin**	*Bacillus cereus, B. mycoides, B. pumilis, B. sphaericus, Bifidobacterium adolescentis, Clostridium perfringens, Escherichia coli, Enterococcus faecium, Eubacterium biforme, Listeria monocytogenes, Paenibacillus alvei, P. azotofixans, P. macerans, P. macquariensis, P. pabuli, P. productus, P. pulvifaciens, P. validus, Peptostreptococcus anaerobius, Pseudomonas aeruginosa, Salmonella enterica, Shigella dysenteriae, Yersinia enterocolitica,*	[24,26]
**Enniatin B**	*Escherichia coli, E. faecium, Clostridium perfringens, Listeria monocytogenes, Pseudomonas aeruginosa, Salmonella enterica, Shygella dysenteriae, Staphylococcus aureus, Yersinia enterocolitica*	[36]
**Enniatin B1**	*Bifidobacterium adolescentis*	[37]

**Table 2:** Products of bacterial metabolism of enniatins and beauvericin.

Mycotoxin	Bacteria	Products of Bacterial Metabolism	Source
EN A	intestinal bacteria	(EN A + K-Ile) ^+^ (EN A + K-HyLv) ^+^	[106]
EN A1	9 bacterial and 22 *S. cerevisiae* strains	(EN A1 + K-Ile) ^+^ (EN A1 + Na) ^+^ (EN A1 + K-Ile) ^+^ (EN A1 + K-HyLv) ^+^	[108]
EN B	6 *Bacillus subtilis* strains	(EN B + K-Val-COOH) ^+^ (EN B + K-Val-H_2_O) ^+^ (EN B + K-HyLv) ^+^ (EN B-2HyLv-H_2_O) ^+^	[109]
EN B	9 bacterial and 22 *S. cerevisiae* strains	(EN B + K-HyLv-Val) ^+^ (EN B-HyLv-2H_2_O-CH_3_) ^+^ (EN B-HyLv) ^+^ (EN B + Na) ^+^ (EN B + K) ^+^	[108]
EN B1	6 *Bacillus subtilis* strains	(EN B1 + K-Ile-Val-H_2_O) ^+^ (EN B1-2Val) ^+^ (EN B1 + K-HyLv) ^+^ (EN B1 + K-Ile) ^+^ (EN B1 + Na) ^+^	[109]
EN B1	9 *Bifidobacterium* and *Lactobacillus* strains and 22 *S. cerevisiae* strains	(EN B1 + K-Val) ^+^ (EN B1 + Na) ^+^ (EN B1 + K-Val-H_2_O-2CH_3_) ^+^ (EN B1+K-Val-HyLv+H_2_O) ^+^	[108]
BEA	*S. cerevisiae* A34	(BEA + Na) ^+^ (BEA + K) ^+^ (BEA + H) ^+^ (BEA–N-Phe-Na) ^+^ (BEA–HyLv) ^+^ (BEA–H_2_O) ^+^ (BEA–HyLv–2H_2_O) ^+^	[110]
BEA	13 *Bifidobacterium, Lactobacillus, Eubacterium,* and *Salmonella* strains	((BEA + Na ^+^) + Phosphatidylcholine)) ^+^ ((BEA + Na ^+^) + Citocoline)) ^+^ (BEA + Na) ^+^	[111]

## Data Availability

No new data were created or analyzed in this study. Data sharing is not applicable to this article.
